# Speciation in progress? A phylogeographic study among populations of *Hemitrichia serpula* (Myxomycetes)

**DOI:** 10.1371/journal.pone.0174825

**Published:** 2017-04-17

**Authors:** Nikki Heherson A. Dagamac, Carlos Rojas, Yuri K. Novozhilov, Gabriel H. Moreno, Rabea Schlueter, Martin Schnittler

**Affiliations:** 1 Institute of Botany and Landscape Ecology, Ernst Moritz Arndt University Greifswald, Soldmannstr. 15, Greifswald, Germany; 2 Engineering Research Institute and Department of Agricultural Engineering, University of Costa Rica, San Pedro de Montes de Oca, Costa Rica; 3 Komarov Botanical Institute of the Russian Academy of Sciences, Laboratory of Systematics and Geography of Fungi, St. Petersburg, Russia; 4 Departamento de Biología Vegetal, Universidad de Alcalá, Alcalá de Henares, Madrid, Spain; 5 Laboratory of Electron Microscopy, Institute of Microbiology, Ernst Moritz Arndt University Greifswald, Greifswald, Germany; Senckenberg am Meer Deutsches Zentrum fur Marine Biodiversitatsforschung, GERMANY

## Abstract

Myxomycetes (plasmodial slime molds, Amoebozoa) are often perceived as widely distributed, confounding to the “everything is everywhere” hypothesis. To test if gene flow within these spore-dispersed protists is restricted by geographical barriers, we chose the widespread but morphologically unmistakable species *Hemitrichia serpula* for a phylogeographic study. Partial sequences from nuclear ribosomal RNA genes (SSU) revealed 40 ribotypes among 135 specimens, belonging to three major clades. Each clade is dominated by specimens from a certain region and by one of two morphological varieties which can be differentiated by SEM micrographs. Partial sequences of the protein elongation factor 1 alpha (EF1A) showed each clade to possess a unique combination of SSU and EF1A genotypes. This pattern is best explained assuming the existence of several putative biospecies dominating in a particular geographical region. However, occasional mismatches between molecular data and morphological characters, but as well heterogeneous SSU and heterozygous EF1A sequences, point to ongoing speciation. Environmental niche models suggest that the putative biospecies are rather restricted by geographical barriers than by macroecological conditions. Like other protists, myxomycetes seem to follow the moderate endemicity hypothesis and are in active speciation, which is most likely shaped by limited gene flow and reproductive isolation.

## Introduction

For the last decades, molecular sequence data were successfully used to disentangle cryptic and emerging species among populations of different groups of organisms. Some examples include green algae [1], marine diatoms [2], fungi [3–4], oomycetes [5] and diptera [6]. Such data have provided a better understanding of the evolution and deep relationships of eukaryotic species, particularly eukaryotic microorganisms commonly recognized as protists [7]. Within protists, myxomycetes are a special case. On one hand, they form visible fructifications, which have been studied for more than 200 years [8]. Therefore, their morphological diversity is better known than for most other groups of protists. On the other hand, myxomycetes are neither pathogenic nor economically important. From this reason the molecular era of myxomycetes started quite late [9], with the first phylogenies constructed only during the last decade [10–14].

Myxomycetes constitute a monophyletic taxon in the supergroup Amoebozoa [11, 15] and are the most species-rich of several groups of protists that, often within independent lineages, disperse by airborne spores released from fruiting bodies [16]. This permitted the development of a morphological species concept, which is applied in all hitherto published monographs of the group, beginning with the first descriptions of myxomycete species by Linnaeus [17], the nomenclatural starting date for the group. Early studies on a few cultivable species, mostly of the Physarales, developed an alternative species concept based on groups of compatible strains (biospecies, [18]). The few morphospecies with numerous strains investigated often split into groups of strains (putative biospecies) that are incompatible with each other; in addition presumably asexual strains were found in culture [19], which casted doubts to which extent the morphospecies concept is able to mirror adequately speciation processes in myxomycetes [20–22]. Recent molecular studies, using mostly the first 600 bp of the ribosomal small subunit rRNA gene (SSU) which is a useful barcoding marker in many protistean lineages [23–24] revealed a high ribotype diversity for most investigated myxomycete morphospecies. A barcoding study on nivicolous species (all dark-spored myxomycetes) from the Caucasus Mountains estimated an average of 1.7 ribotypes per morphospecies [25]. A survey focusing on lignicolous species (all bright-spored, the second major group of myxomycetes) found a figure of 2.9 [26]. Similar figures for the number of ribotypes per morphospecies have been found in studies on singular morphospecies with multiple accessions screened: Fiore-Donno et al. [11] found 3 and 7 ribotypes for *Lamproderma puncticulatum* and *L*. *columbinum*, respectively, Aguilar et al. [27] reported two morphological differentiable groups within *Badhamia melanospora* with 14 and 23 ribotypes, Leontyev et al. [28–29] reported 24 ribotypes for *Tubifera ferruginosa* s. str., with some of these corresponding to differentiable morphological characters, and Feng & Schnittler [30] reported 18 ribotypes for *Trichia varia*. Studies employing multiple markers for *Trichia varia* [30] and *Meriderma* spp. (formerly *Lamproderma atrosporum*, [20]) revealed groups of ribotypes that seem to be reproductively isolated from each other, constituting putative biospecies. If these relationships are the rule, the number of nearly 1 000 species described worldwide at a morphospecies level [31] would inflate by a factor between 2 and 10 at the biospecies level. To look if such putative biospecies differ as well in their ecological niches, the morphologically clear cut species *Hemitrichia serpula* (Scop.) Rost. (Trichiaceae) was chosen for this world-wide biogeographic study.

**Fig 1:**
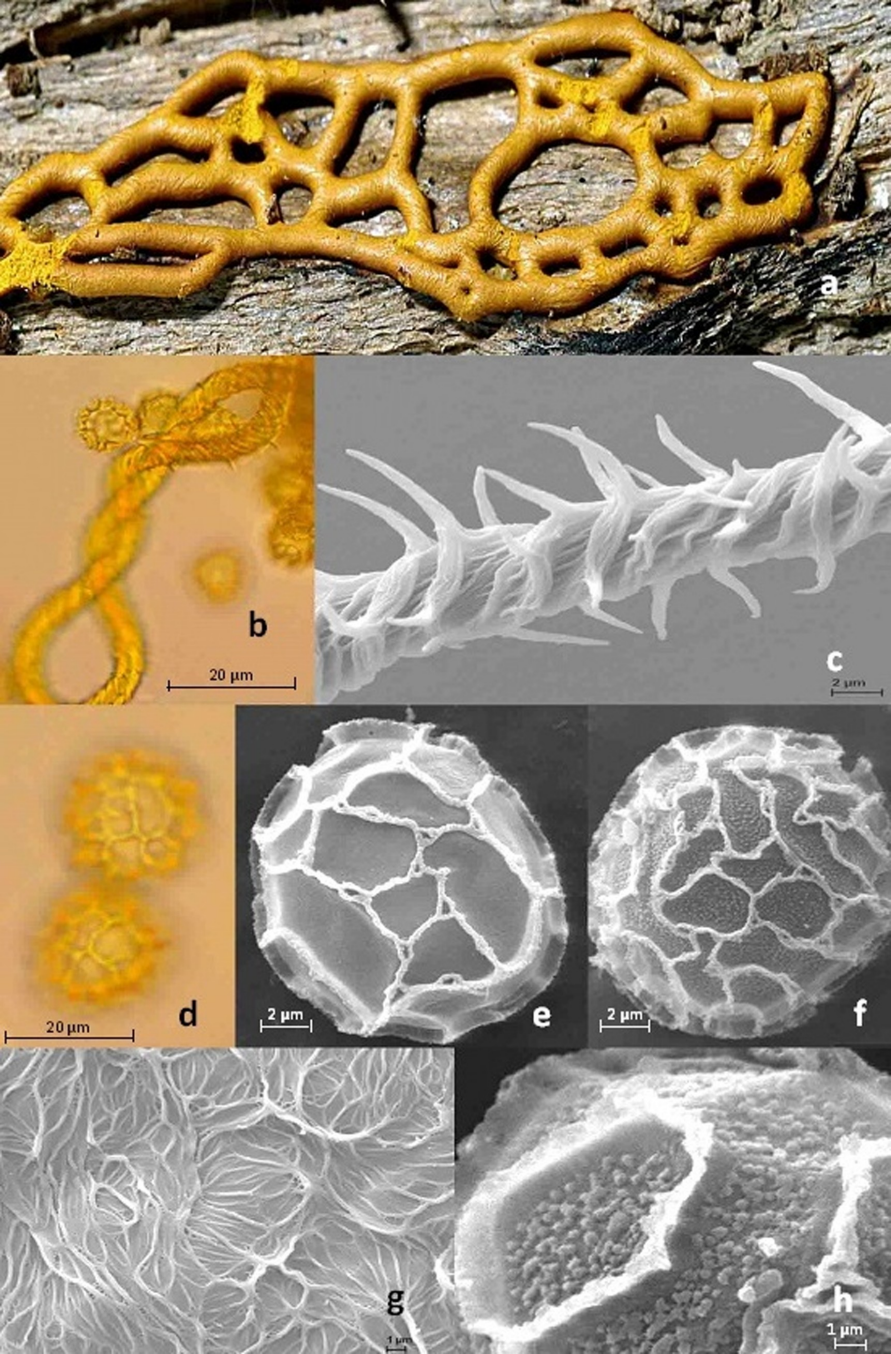
**Morphology of *Hemitrichia serpula*:** a. mature fruiting body, b-c. capillitium of var. *serpula* (specimen M754) as seen in (b) light microscope and (c) scanning electron microscope (SEM), d. spore morphology of var. *serpula*, e-f. SEM micrograph of spores for (e) var. *serpula* (LE297865) and (f) var. *parviverrucospora* (sc28101), g. SEM of the internal linings of the peridium, var. *parviverrucospora*, h. close-up, showing the internal warts between the reticulations of var. *parviverrucospora* (sc28065).

*Hemitrichia serpula* (Fig 1) is a widespread myxomycete characterized by plasmodiocarps forming an unmistakable golden yellow reticulum with a spinulose capillitium and coarsely banded-reticulated spores. In spite of this distinct habit, morphological characters vary to a certain extent within this morphospecies, which led to the description of several varieties. As for virtually all myxomycetes, the mechanisms behind such variability (truly genetic or a caused by phenotypic plasticity) are still unknown [32]. Nannenga-Bremekamp and Yamamoto [33] described var. *tubiglabra* from dead mossy wood found in Nepal, based on a more robust capillitium (8–9 μm in diam.) with smooth spirals and spores with a coarser reticulum. Cavalcanti & Mobin [34] named var. *piaueinsis* from Brazil, noting the more scattered and shorter spines of the capillitium and a finer and more regular reticulation of the spores. Finally, Lizarraga *et al*. [35] published var. *parviverrucospora* based on a faint reticulation visible in SEM that covers the usually smooth areas between the reticulum of the spores. This variety was later elevated to species rank [36]. These taxonomic studies triggered the first questions this study intends to answer: Do these morphological differences hint towards the existence of cryptic species within *Hemitrichia serpula*? Second, do they coincide with molecular sequence data?

Finally, the study was set up to evaluate the biogeographic hypotheses “everything is everywhere” against the concept of “moderate endemicity”, combining a ribotype phylogeny with environmental niche modeling (ENM). Based on morphological data, many species of myxomycetes seem to be cosmopolitan (e.g., *Barbeyella minutissima*, [37]). If restrictions appear, temperature seems to be a factor (e.g. *Ceratiomyxa morchella*, [8]). Not surprisingly, long-distance dispersal of myxomycete spores was demonstrated by Kamono *et al*. [38]. The potentially effective dispersal combined with an ephemeral life style (suitable habitat conditions have to be realized only for limited periods of time in small spatial niches) let myxomycetes appear as a prominent example for the ubiquity hypothesis of protist biogeography that “everything is everywhere” (EiE). However, the environment selects, and therefore patchy distribution patterns are not only due to insufficient knowledge but can be expected by intermittent occurrence of suitable habitats, as demonstrated for *Barbeyella minutissima* [37]. Myxomycete spores are typically between 8 and 12 μm in diameter; terminal velocity for falling in air seems to follow Stokes law [39]. This should result in leptokurtic dispersal curves, as found for fungal spores [40] or pollen grains [41]. Hence geographic barriers (like oceans) can be expected to restrict gene flow between populations of a widely distributed myxomycete species. This may cause allopatric speciation and is more in accordance with the “moderate endemicity” model. Supporters of the ubiquity hypothesis assume a lower global diversity among free living protists since their distribution is cosmopolitan [42–43] but the proponents of the moderate endemicity hypothesis assume a higher global diversity and thus a significant amount of hidden diversity [44–45]. The differences found between ribotypes of *Badhamia melanospora* in the Old and New World [27] seems to advocate the latter hypothesis, assuming a moderate level of endemicity for protists [46–47]. However, speciation processes in myxomycetes are virtually not studied. *B*. *melanospora* may be a special case, as this species is specialized on decaying tissues of succulent plants and should thus be limited to arid regions, whereas *Hemitrichia serpula* grows on all kinds of litter and is well known for its global distribution (www.discoverlife.org). With its conspicuous fructifications, it is a kind of flagship species within myxomycetes and seems to be well suited to test both hypotheses.

Environmental niche models (ENM) may help to decide if “phylogroups”, which are likely to constitute cryptic species within a morphospecies, are limited by ecology (environmental constraints) or by geography (dispersal barriers). These models rely on numerical tools that combine observations of species occurrence or abundance with environmental parameters to calculate potential distribution ranges [48]. Rojas *et al*. [49] used this tool to predict the distribution probability of five common myxomycete species in Costa Rica. In this study, we investigated to which extent phylogroups in *H*. *serpula* may be restricted by dispersal barriers or by environmental constraints.

## Materials and methods

### Specimen acquisition

Lowland forests from four major land masses (Asia, Europe, North and South America) were surveyed for *Hemitrichia serpula* between 2012 and 2015. Additionally, specimens from other myxomycete collectors were obtained, resulting in a total of 135 herbarium specimens coming from 43 localities that were used in this paper. The holotype specimen (AH24440 from Mexico) and an isotype specimen (AH43993 from Argentina) of *H*. *serpula* var. *parviverrucospora* were obtained from the herbarium collection of G. Moreno. Collections from Costa Rica were made under research permit 012–2014-ACAT from MINAE and collections from the Philippines were made under the gratuitous permit issued by the Department of Environment and Natural Resources (DENR). Information about the localities, determination results and Genbank accession numbers (KY100981–KY101149) of the specimens used in this study are listed in S1 File.

### Morphological evaluation

Specimens of *H*. *serpula* were analysed independently using scanning electron microscopy (SEM), examining especially internal structures within the spore mesh. According to the origin of the studied collections, three different instruments were used (DSM-950, Carl Zeiss, critical point drying, University of Alcalà de Henares, Spain, 11 specimens; JSM-6390 LA, Komarov Botanical Institute RAS, St. Petersburg, 22 specimens; EVO LS1, Carl Zeiss, Imaging Center, Greifswald University,100 specimens).

### DNA extraction, amplification and sequencing

A part of a plasmodiocarp that equals 10–15 separate sporocarps of *Trichia varia* was transferred into a sterile 2 ml safe-lock Eppendorf tube with coarse sea sand, cooled to −80°C for 30 minutes, and homogenized manually using a plastic pestil. DNA was extracted with the E.Z.N.A. Plant DNA Kit (Omega Bio-Tek, Georgia, USA), following the manufacturers protocol.

Amplification of the first part of the nuclear small subunit ribosomal RNA gene (SSU) was conducted for 135 samples of *H*. *serpula* using two primers designed for this study, named S1A (CTGGTTGATCCTGCCAGAAT) and SRHem1 (CGGGGTTTAAAGGTCCCC, all primer sequences written in 5'-3' direction). A total volume of 25 μl, adding 5 μl colored reaction buffer (5x Mango-Taq^TM^), 1.7 μl MgCl_2_ (Bioline, 50 mM), 0.5 μl dNTPs, 0.625μl of each primer, 0.2μl (1U / μl) of Mango-Taq^TM^ DNA Polymerase, and 4μl of template DNA was used and adjusted with ddH_2_O. PCR included 2 min at 95°C, 39 cycles (30s at 95°C, 30s at 52°C, 1 min at 72°C) and 5 min at 72°C.

Three specimens (sc28076, sc28090, sc28128; all from Costa Rica) displayed heterogeneous SSU sequences. To ascertain these results, the PCR was repeated. In all cases, the two overlaid sequences could be disentangled, since they matched already existing ribotypes. An exception was one allele of specimen sc28076, which represented a new, slightly deviating ribotype. For all further analyses, the heterogeneous sequences were coded like two alleles, and both were included in phylogenetic analyses.

Partial sequences of the protein elongation factor 1 alpha (EF1A) were obtained from the last part of the gene (downstream of the spliceosomal intron, covering amino acids 141–393, 243 triplets) referring to the sequence from *Physarum polycephalum* (GenBank AF016243, [50]). We amplified sequences from 30 samples using the newly designed primer pair EMyxF4 (CTYGGTGTGAARCARATGATYGT) and EMyxSF1rmod (CCTTCCAAGCCCATGTGYGT). The reaction mix included 5μl colored reaction buffer (5x Mango-Taq^TM^), 0.7 μl MgCl_2_ (Bioline, 50 mM), 0.5 μl dNTPs, 1.5 μl of each primer, 0.2 μl (1U / μl) of Mango Taq DNA Polymerase, and 4μl of template DNA, adjusted to a total volume of 25 μl with ddH_2_O. Cycle conditions were 2 min at 94°C, 45 x (30s at 94°C, 30s at 52°C, 1 min at 72°C) and 5 min at 72°C. All products were purified with the SureClean kit (Bioline) and analyzed with an ABI 3730 sequencer.

### Sequence alignment and phylogenetic analyses

The generated sequences were initially aligned automatically, using default settings of the software MUSCLE [51] executed in Mega 6.0 [52] and, if necessary, corrected manually. Alignments including 453 sites (SSU, S2 File) and 729 sites (EF1A, S3 File) were used for further phylogenetic analyses. All 135 investigated specimens were sequenced for SSU, and a total of 30 specimens representing each major clade in the SSU phylogeny were selected to generate EF1A sequences. Phylogenetic trees were constructed using Bayesian Interference (BI), MrBayes 3.2.5 [53] with the GTR + I + γ model of substitution, the gamma distribution being approximated by eight categories. The MCMC search was run with 4 chains for 1 million generations with sampling every 100 generations. Maximum Likelihood (ML) trees were calculated with RAxML [54] using the model GTR+GAMMA for nucleotide substitution, the rapid hill-climbing (–fd) option and the rapid bootstrap algorithm (–fa) with 1 000 bootstrap replicates. The resulting “.tre” output file was edited using FigTree v. 1.4.2 [55]. A sequence from a specimen of *Trichia varia* (GenBank JX481344) was used as outgroup in the phylogenetic analyses. To calculate a parsimonious network for ribotypes with a 95% of coherence according with a set of possible outcomes based on coalescent theory [56], a gene genealogy of all unique ribotypes separated by mutational steps was created using the software TCS v.1.21 [57]. TCS calculates the probability that pairs of ribotypes are similar for all combinations of ribotypes and then joins the most similar ribotypes together into a network where their combined probability is >95%. Therefore, the resulting network will remove divergent ribotypes whose true genealogy may be concealed by homoplastic characters [58].

### Genetic analyses

To estimate the exhaustiveness of the survey, a ribotype accumulation curve was constructed using EstimateS (Version 9.1, 100 randomizations, [59]). In accordance with Unterseher *et al*. [60] the Chao1 estimator [61] was chosen and calculated using the “default settings” of EstimateS.

Correlation between pairwise combinations of genetic and geographic distances was tested for significance by a Mantel test. Two matrices were constructed, first for pairwise genetic distances from the 480 bp multiple sequence alignment of only the specimens with homogenous SSU sequences, applying the maximum composite likelihood model implemented in MEGA 6.0. The second matrix includes all geographic distances between the collection sites of any two specimens, which were computed with an Excel macro for the Vincenty formula [62]. Correlations and the Mantel test with 999 iterations were calculated with the ExtraStats function in PopTools v. 3.2.5 [63].

To detect differentiation of ribotypes among geographical groups obtained in this study, an analysis of molecular variance (AMOVA) was performed using Arlequin 3.5 [64] with a significance based on 10 000 permutations. An analysis of polymorphic sites that generated the diversity of the ribotypes, nucleotide diversity, and average number of nucleotide differences was performed using DnaSP v. 5.10 [65]. Furthermore, the same software was used to calculate the DNA divergence between major phylogenetic clades that resulted in a pairwise matrix showing the mean number of nucleotide difference and nucleotide substitution per site between clades.

### Historical biogeography

An event-based ancestral area reconstruction suggesting an explicit model of processes that possibly explains the geographic distribution of our generated SSU tree was constructed by defining distribution areas based on the three major sampling regions: (A) Northern temperate zone (Far East of Russia, Europe and Eastern North America), (B) Paleotropics (Southeast Asia, Africa), and (C) Neotropics (Carribean region, Central and South America). We ran S-DIVA analyses using RASP v.3.01 (Reconstruct Ancestral State in Phylogenies; [66]) to infer a probable biogeographic history of *H*. *serpula* ribotypes based on the phylogeny constructed from partial SSU data. In S-DIVA, the frequencies of an ancestral range at a node in the ancestral reconstructions are averaged over all trees, and each alternative ancestral range at a node is weighted by the frequency at which the node occurs or by some other measure of support for the node [67]. The previously produced 10 001 trees in MrBayes 3.2.5 and the distribution matrix of each ribotypes were loaded into the software. The maximum number of ancestral areas at each node was limited at two; maximum reconstruction was set to 100, and maximum reconstruction for the final tree was set to 1 000.

### Species distribution modeling

For each of the three most important clades in the SSU phylogeny distribution was modelled based on world bioclimatic grid data (rasters) downloaded from the BIOCLIM dataset (http://www.worldclim.org/bioclim). Initially, grid data for all 19 bioclimatic variables were tested for all three clades using the MaxEnt (Maximum entropy) v3.3.3k software [68] to choose the best combination of variables able to explain at least 70% of the variability in the model. According to this criterion bioclimatic variables 3, 7, 12 and 15 (Isothermality, Annual temperature range, Annual precipitation and Seasonality of precipitation) were chosen and all models were created using these variables only. In addition, elevation data were included in the models. Finally, all models were ran on MaxEnt, using standard settings, applying a bootstrap replicated run type and 500 maximum iterations on 50 different runs per biotype. To assess the accuracy of the discriminatory capacity (sensitivity for true positive and specificity for true negative) of the generated niche models, each model was evaluated using the receiver operating characteristic (ROC) analysis that resulted to the area under the curve (AUC) values. This value translates to the chance that a randomly selected presence has a greater predicted probability of occurrence than a randomly chosen absence [69]. The final models were also subjected to the analysis of variable contributions. For visualization, the rasterized output data were imported into ARCMap v10.2 to reclassify values for probability of occurrence under a particular model into a heat map.

## Results

### Morphological and molecular diversity

Fig 1 shows the morphological structures for peridium, capillitium and spores of *Hemitrichia serpula*. All except two of the 135 specimens were examined by SEM, with 74 determined as var. *serpula*, 58 as var. *parviverrucospora*, and 1 as var. *tubiglabra*; represented by 26, 14, and 1 ribotype, respectively. Most ribotypes could be uniformly attributed to either var. *serpula* (25 ribotypes from 70 specimens) or var. *parviverrucospora* (13 ribotypes from 54 specimens). However, two ribotypes included specimens from both varieties: r3 (15 specimens var. *serpula* / 4 var. *parviverrucospora*) and r28 (4 var. *serpula* / 19 var. *parviverrucospora*). The ribotype of the single specimen of var. *tubiglabra* (r26) is shared with three other specimens determined as var. *serpula*.

The alignment of partial SSU sequences comprised 480 positions; of these 96 were polymorphic, including 17 variable singleton sites and 79 parsimony informative sites. Counting the alleles of three heterogeneous ribotypes separately for each allele, 40 ribotypes were found for 138 sequences from 135 specimens. According to an accumulation curve constructed for the ribotypes, 52% (Chao 1 estimator) of all ribotypes to be expected were found.

**Fig 2:**
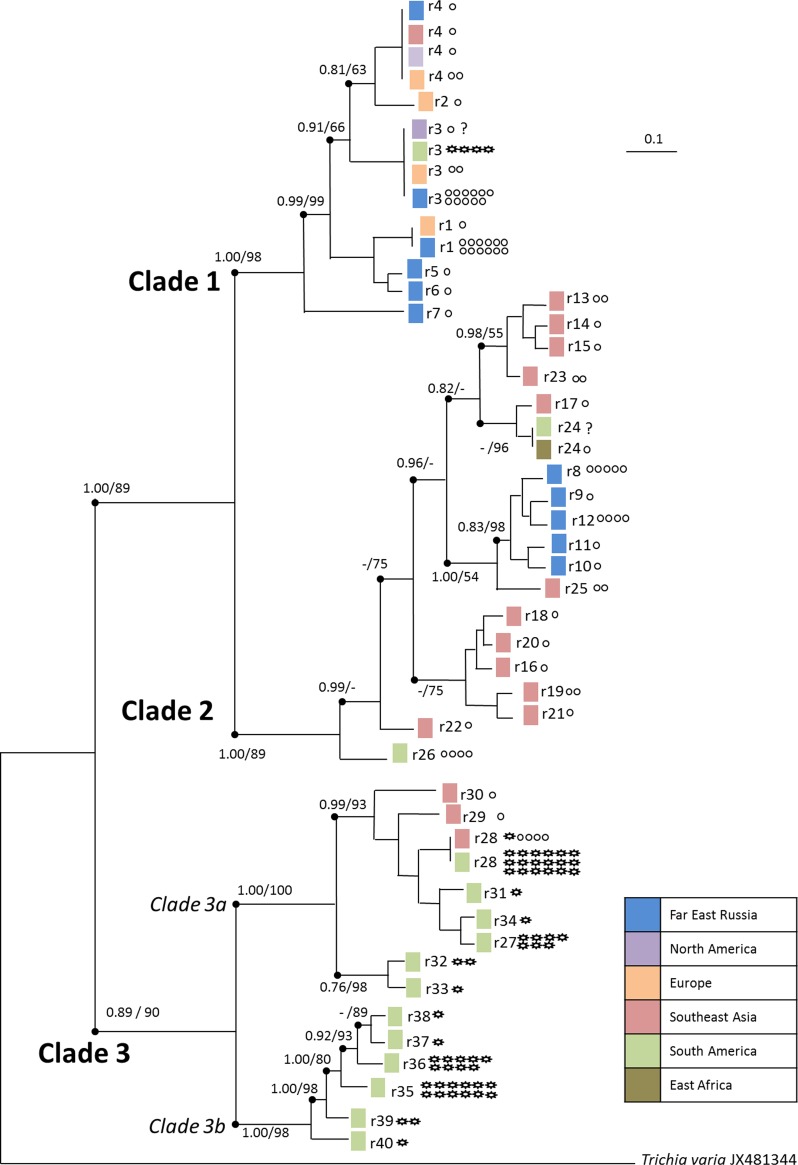
Rooted consensus tree based on the 50% majority rule of Bayesian interference for partial SSU sequences for 40 ribotypes from 135 specimens of *Hemitrichia serpula*. Shown are Bayesian posterior probabilities >0.70 and support values >50 for a corresponding tree calculated with RAxML (all branches indicated by a dot). Colored squares indicate the origin of the specimens. The ribotype number and the morphology of the respective specimens are indicated by smooth (var. serpula) and spiny circles (var. parviverrucospora). Question marks indicate the two specimens with undetermined spore morphology. Scale bars represent evolutionary distance as changes per site.

Using *Trichia varia* as outgroup, Bayesian interference and Maximum likelihood analyses of partial SSU sequences resulted in identical tree topologies differentiating three major clades (Fig 2), yet with different figures for bootstrap / posterior probability support. Clade 1 was composed of 41 sequences (36 came from areas in the temperates and 5 from the tropics) and 7 ribotypes. The more divergent clade 2 included 34 sequences (17 originate from the old world tropics, 12 from the Russian Far East and 5 from South America) with 19 ribotypes. Clade 3 splits into two prominent subclades (3a and 3b) those altogether comprising 67 sequences with 14 ribotypes, all coming from tropical regions (Table 1).

**Table 1:** Summary of the polymorphic site analysis for each clade (putative biospecies) generated from DNAsp v.5.10 (see Fig 2 for corresponding phylogeny)

	Clade 1	Clade 2	Clade 3a	Clade 3b
Number of sequences	41	33	38	26
Number of polymorphic site	13	44	61	15
Total number of mutations	13	47	64	17
Total number of singleton mutations	11	4	55	12
Average number of nucleotide differences	1.244	12.239	4.312	2.209
Nucleotide diversity	0.00275	0.02726	0.00950	0.00489
Haplotype diversity	0.684	0.913	0.380	0.683

Clades 1 and 2 included var. *serpula* (66 of 70 specimens, spores with smooth intermesh areas, Fig 1E); with the exception of four specimens from Costa Rica (sc28033, sc28034, sc28090 and sc28128). Most (57 of 63 specimens) of clade 3 fit the description of var. *parviverrucospora* (spores with faintly warted intermesh areas, Fig 1F). Specimens used to describe var. *parviverrucospora* (ribotypes r33, isotype AH43993 from Argentina and r39, holotype AH24440 from Mexico) grouped in subclades 3a and 3b, respectively.

The three specimens showing heterogeneous SSU sequences, all from Costa Rica, represented a mixture of ribotypes r3+r35 (sc28090) and r3+r28 (sc28128); all these ribotypes were as well recorded in homogeneous state in the population. The remaining specimen sc28076 was composed of ribotype r39, which is the ribotype of the type specimen of var. *parviverrucospora* (AH24440 from Mexico) and a new ribotype r40, deviating in seven positions.

**Fig 3:**
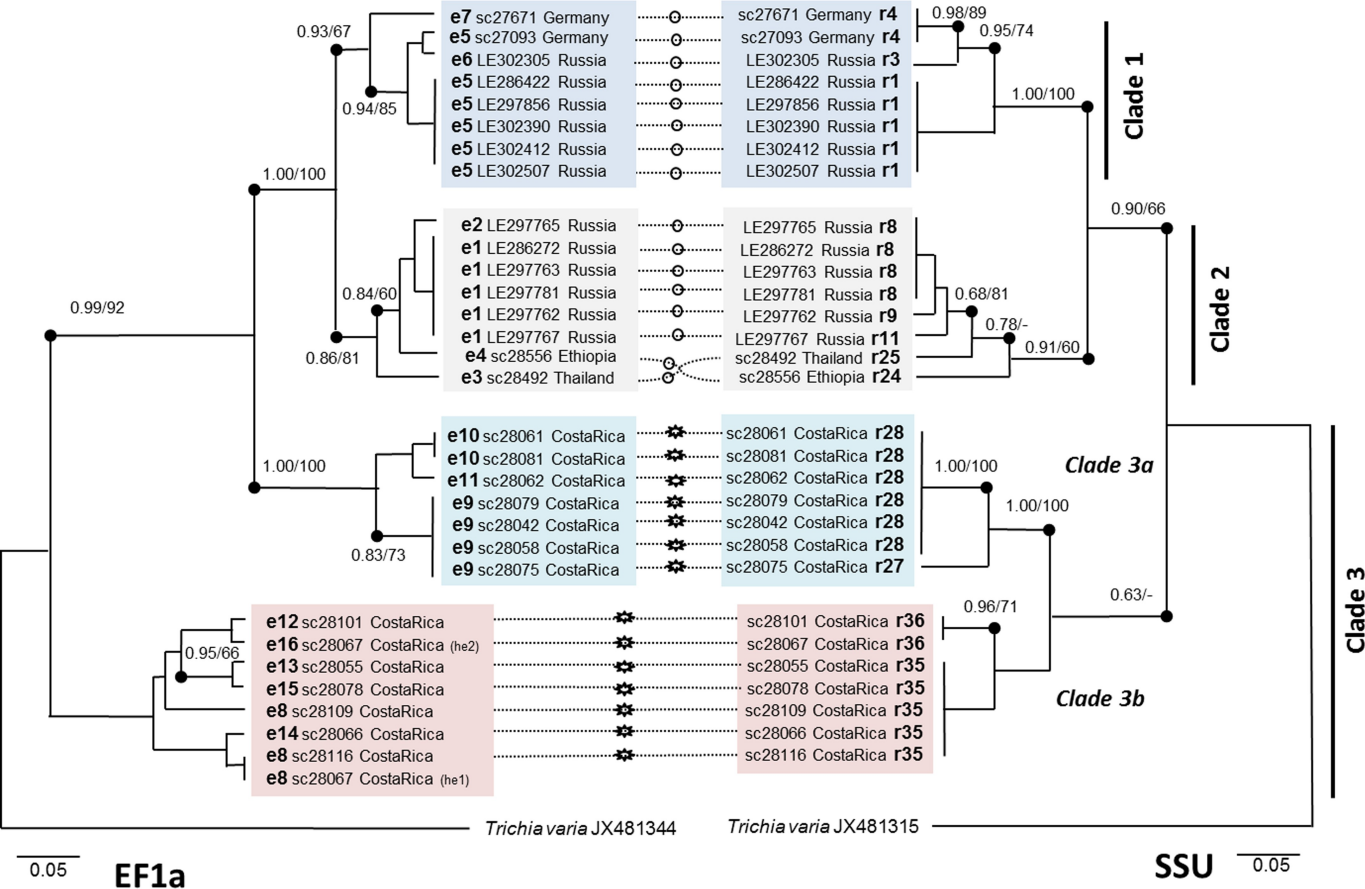
Phylogenetic analysis showing a mirrored image comparing tree topologies for partial SSU and EF1A sequences for 30 specimens. Bayesian posterior probabilities >0.70 and RAxML support values >50 are indicated (nodes with dots). Scale bars represent evolutionary distance as changes per site. Ribotype (r) and EF1A genotype numbers (e) are shown for both markers. Dotted lines connect sequences from the same specimen, with symbols for var. *serpula* (smooth circles) and var. *parviverrucospora* (spiny circles) in the middle.

EF1A as a second independent marker differentiated the same major clades within 30 selected specimens, yet with a somewhat different topology. Fig 3 shows the mirrored trees of partial SSU and partial EF1A sequences. All clades displayed exclusive combinations of EF1A genotypes and SSU ribotypes (clade 1: 3 and 3, clade 2: 4 and 5, subclade 3a: 3 and 2, subclade 3b: 6 and 2). All EF1A genotypes and SSU ribotypes were limited to a single clade/subclade, with the exception of the EF1A genotype 8, which showed a single clear heterozygosity (specimen sc28067), constituting a mute mutation in the third base of a triplet coding for Leucin. Specimens from heterogeneous or morphologically "incorrect" ribotypes (e.g., r3, r29, r30) did not show any successful amplifications for EF1A.

**Fig 4:**
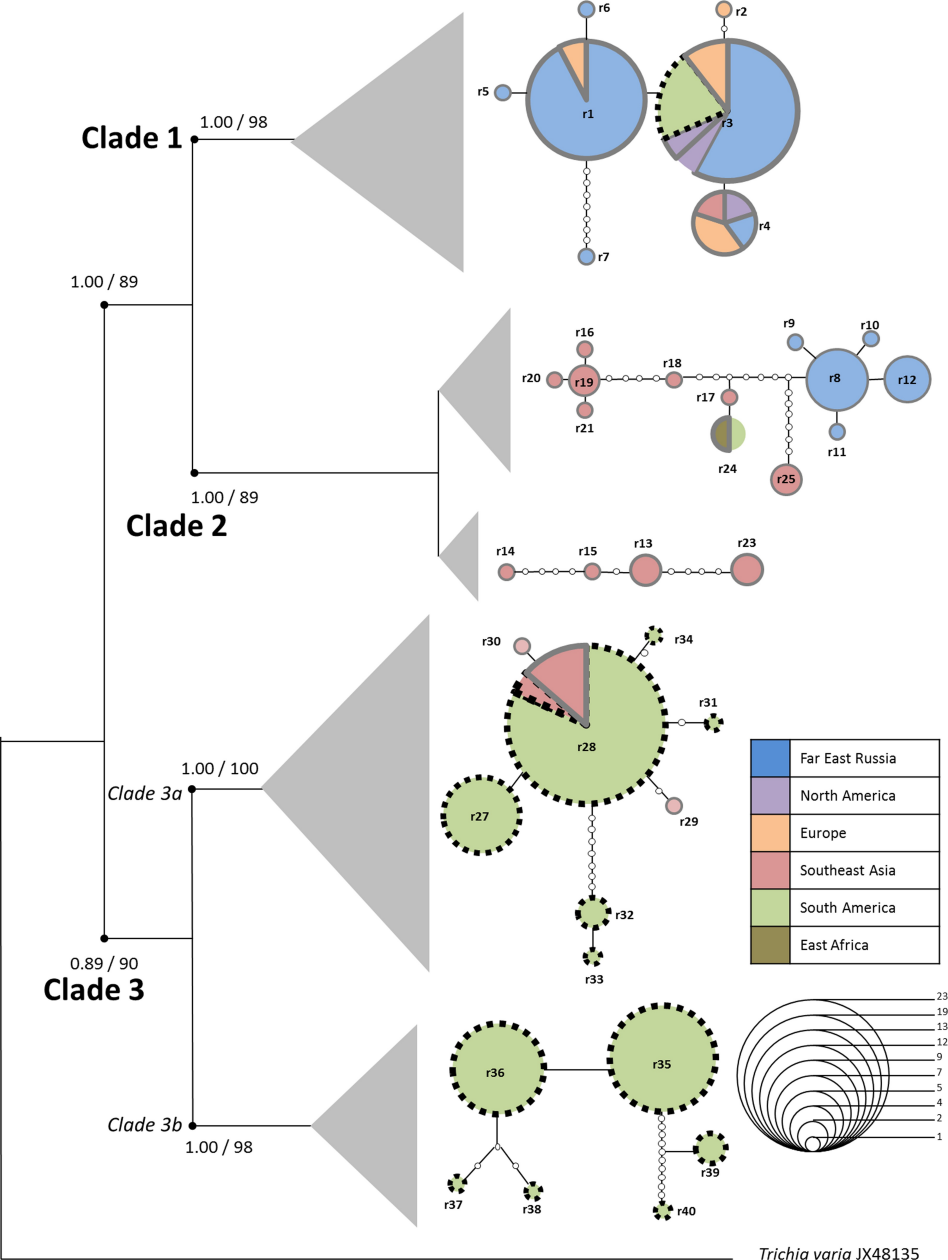
Statistical parsimony ribotype network representing genealogical relationships among 40 ribotypes estimated by TCS superimposed on a Bayesian interference tree. Grey triangles are sized relative to the number of specimens per network. Line segments represent mutational steps between alleles. Circles are scaled in proportion to the number of sequences represented by each ribotype. Small circles between ribotypes indicate hypothetical transitional ribotypes. Colors designate the origin of the specimen. Morphotypes displayed by specimens showing the respective ribotype are indicated by smooth (var. *serpula*) or broken lines (var. *parviverrucospora*).

The ribotype network (Fig 4) was highly compatible with the constructed phylogenetic trees. Due to the large distance between the clades (Table 2) the software could not connect all sequences to a single network. Instead, every clade or subclade formed its own network. An exception was the most diverse clade 2, resulting in two separate networks. In addition, the statistical parsimony algorithm employed in TCS failed to include its two most distant ribotypes of clade 2 (r22, a singleton and r26, four specimens) into a network.

**Table 2:** Pairwise matrix showing the average number of nucleotide differences between clades (lower left) and the mean number of nucleotide substitutions per site between each clades (upper right)

	Clade 1	Clade 2	Clade 3a	Clade 3b
**Clade 1**	-	0.06542	0.10809	0.08816
**Clade 2**	29.244	-	0.11606	0.09505
**Clade 3a**	48.640	51.646	-	0.07852
**Clade 3b**	39.494	42.106	35.335	-

### Ancestral area reconstruction

**Fig 5:**
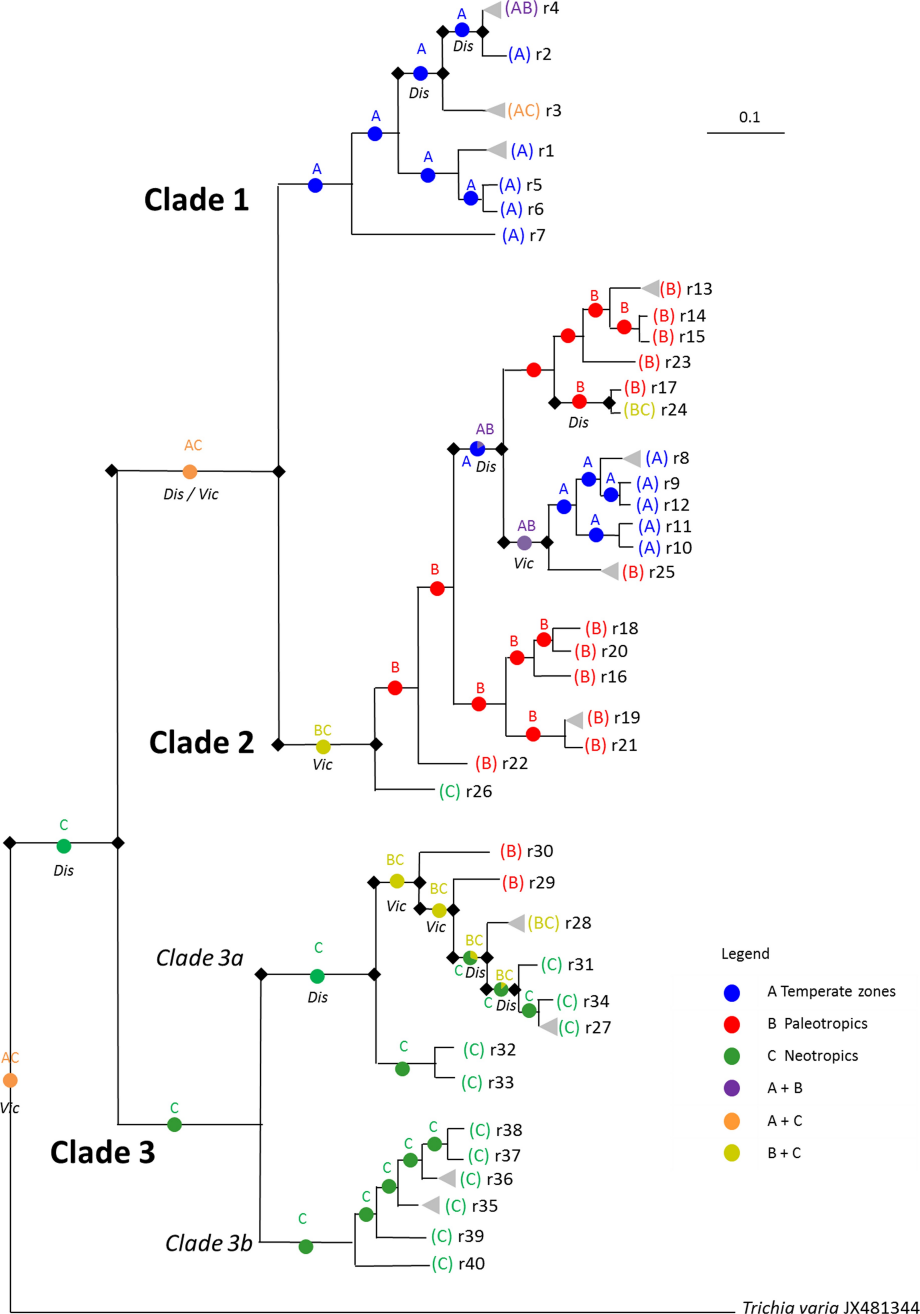
Hypothesized event-based (Vic = vicariance; Dis = dispersal) ancestral area reconstruction of *H*. *serpula* ribotypes as inferred by S-DIVA analysis of RASP. Pie charts at the nodes give relative frequencies of the ancestral-area reconstruction. Grey triangles indicate ribotypes represented by multiple specimens.

The S-DIVA analysis (Fig 5) proposed 9 dispersal and 6 vicariance events to account for the present distribution of ribotypes. According to this analysis the center of origin for *H*. *serpula* lies in the Tropics of the New World; subsequent dispersal events led to the separation of the three major clades. The ancestral area for clades 1 and 2 may be situated in the temperate zone and the New World Tropics, respectively; further dispersal and vicariance events caused the current distribution of the ribotypes within the clades.

### Population structure

The world population of *H*. *serpula* is geographically structured: pairwise geographic and genetic distance matrices are correlated with each other (R = 0.4667). According to a Mantel test, this value is significant, since it falls clearly out of the 95% confidence interval (-0.0134 to 0.0211) calculated for 999 matrix permutations. Accordingly, one can expect that geographical separation explains a substantial proportion of the genetic variation among the world population of *H*. *serpula*.

However, the analysis of molecular variance among the proposed distribution areas (Table 1) showed higher variation within a region (temperate zone / Neotropics / Paleotropics) than among these regions. These figures reverse if molecular variance within / among the major clades 1–3 is analyzed: now two thirds of the variation is among clades.

### Species distribution modeling

**Fig 6:**
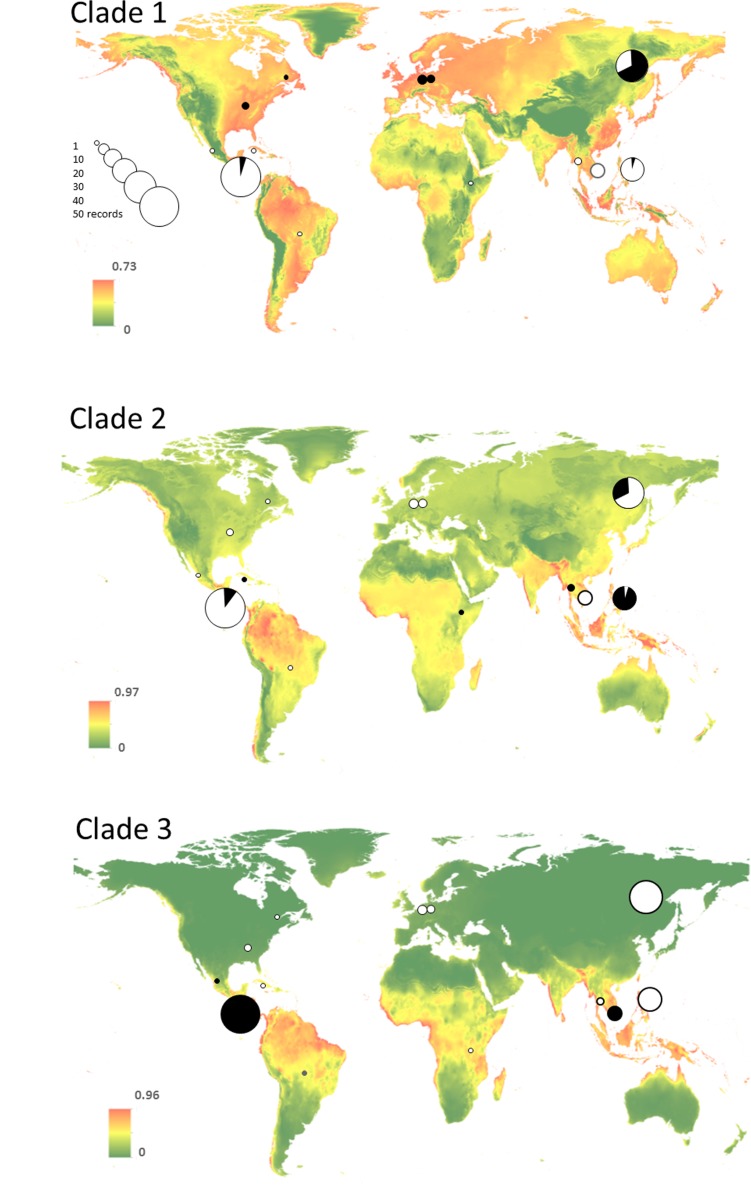
Probability-based environmental niche models for the three major clades in a ribotype phylogeny of *Hemitrichia serpula* calculated with the MaxEnt algorithm. Circles are located over areas where specimens were collected; their size is scaled according to the number of specimens collected in an area. Black filling of the pie diagrams indicates the proportion of specimens belonging to the respective clade. The underlying heat map shows the likelihood of occurrence for the respective clade.

Niche modeling based on the recorded species occurrence was carried out separately for each major clade (subclades 3a and 3b were pooled) with the MaxEnt algorithm (Fig 6). If the area under the curve (AUC) is taken as a measure of the predictive value of the model, quality improved from clade 1 to clade 3 (0.789, 0.888, 0.921). Clade 2 apparently has a broader distribution range than clade 3. Considering a changing environmental scenario, the model consistently showed that clade 3 ribotypes seem to be limited to tropical regions. For all three clades, elevation was the most important variable. The second-most important variable changed with the time scenario: for current climatic conditions it was seasonality of precipitation, for the future scenario it was overall annual precipitation, for the past scenario it was annual temperature range.

## Discussion

The accurate circumscription of species is an essential prerequisite for any diversity assessments and biogeographical studies, which is most often based on morphological criteria. Applying a morphological species concept to myxomycetes poses two obvious difficulties. First, fructifications develop not out of a growth process but by rearrangement of the biomass of the plasmodium within a short period of time, typically hours to days [16]. Therefore, extreme weather events during the short time of sporocarp development may easily cause aberrations in morphological characters [32]. Sporocarps are not actively living but contain only spores as dormant stages. As long as spores can be dispersed, these structures can exhibit a high phenotypic plasticity without being penalized by a lower fitness. Second, the fructifications show only a limited number of morphological characters. Therefore, it cannot be expected that every geno- or ribotype will be recognizable by a unique combination of characters. In addition, none of the markers currently established for myxomycetes codes for any morphological character.

So far, all studies investigating SSU sequences for numerous accessions of a myxomycete species resulted in multiple ribotypes; an apparent exception was only *Hemitrichia calyculata* (one ribotype shared by 52 accessions, but these are all from one area [26]). Therefore, a nearly cosmopolitan species like *Hemitrichia serpula* is likely to consist of numerous different ribotypes, which can be used to test hypotheses on protist biogeography.

### Ribotypes and morphological varieties in *Hemitrichia serpula*

Only two of the four described varieties of *Hemitrichia serpula* occur commonly, and the diagnostic character telling them apart is the absence (var. *serpula*) or presence (var. *parviverrucospora*) of the internal warts that can be seen inside the reticulum of ridges covering the spores (Fig 1). Most but not all of the specimens grouping in clades 1 and 2 have the morphology of var. *serpula*, whereas most specimens of clade 3 were assigned to var. *parviverrucospora* (Fig 2). The two other described varieties are very rare; they differ in capillitial characters (shorter spines, var. *piaueinsis* / rare to missing spines, var. *tubiglabra*) and spore ornamentation (finer / coarser reticulation). We were only able to obtain a single specimen from var. *tubiglabra*, and its ribotype (r26) is identical to this of three other specimens clearly assignable to var. *serpula* (clade 2). This indicates that the var. *tubiglabra* does not bear any taxonomic value. This situation may be not uncommon for rare morphotypes that differ from their common counterparts only by the lack or malformation of a character (like the capillitial spines), which is likely to be caused by environmental alterations during development [32]. An examination using molecular methods may help to avoid formal description of such morphotypes.

### Cryptic speciation in *H*. *serpula*

The phylogeny based on partial SSU sequences clearly revealed three distinct clades (Fig 3) for *Hemitrichia serpula*, with clade 3 splitting again into two subclades. In myxomycetes, nuclear (18S) SSU genes reside with multiple copies on extrachromosomal units and show non-Mendelian inheritance (see discussion in [30]). To confirm this topology, selected specimens were sequenced with an independent second marker. EF1A is a nuclear single copy gene located on regular chromosomes and is expected to show Mendelian inheritance. Each of the four phylogroups (clades 1, 2, and subclades 3a and 3b) was characterized by a unique combination of SSU and EF1A genotypes (Fig 3). This pattern is similar to the results found for *Trichia varia* [30], where three markers, SSU, EF1A and the mitochondrial cytochrome oxidase gene (COI) displayed as well clades with unique combinations of the genotypes. All these markers are independent from each other and should thus freely recombine in a sexual population. Therefore, the absence of recombination among the clades is explained best by the assumption of reproductively isolated units, i.e. biospecies in the sense of Clark and Haskins [19]. Feng *et al*. [20] provide a discussion of the biospecies concept and possible reproductive modes in myxomycetes on the example of the genus *Meriderma*, where as well several reproductively isolated, putative biospecies were found with SSU and EF1A markers. In the case of this study, the reproductive isolation is additionally underpinned by the distribution of the two varieties among clades (var. *serpula* dominates clades 1 and 2, var. *parviverrucospora* subclades 3a and 3b), with none of the phenotypic characters distinguishing the varieties coded by any of the investigated markers.

### Ongoing speciation?

However, exceptions proof these rules and indicate that reproductive isolation is not complete for *H*. *serpula*. First, four of seventeen specimens belonging to the common ribotype r3 (clade 1) showed the “wrong” phenotype (var. *parviverrucospora*, Fig 2); likewise four, one and one specimens of ribotypes r28, r29, r30 (all subclade 3a) belong to var. *serpula*. Second, the single heterozygous EF1A genotype (e7) occurred in two clades (1 and 3b). Third, three specimens from the most diverse population (Costa Rica) displayed heterogeneous SSU sequences, representing in part apparent crosses between representatives of different clades (1 x 3a, 1 x 3b, 3b x 3b). Such heterogeneous SSU sequences are rare since the crosses between two ribotypes are usually homogenized by gradual elimination of one ribotype during plasmodium formation [70], but were found as well in *Trichia varia* [30].

These inconsistencies may be explained best by recent dispersal events and subsequent hybridization between amoebae belonging to usually separated clades. This can happen due to the capacity of myxomycete spores to disperse over long distances [38]. A similar case was pointed out in another phylogenetic study of the myxomycete genus *Meriderma*, where a single specimen showed incongruence between SSU and EF1A phylogroups [20]. As indicated by the geographic origin of ribotypes from different clades and by the results of a Mantel test, geographic separation within ribotypes of *H*. *serpula* occurs but is incomplete. This is corroborated by the AMOVA (Table 3) results for the three investigated regions (temperate zones, Neo-, and Paleotropics) which indicated higher variation within a region than among the regions. This evolutionary scenario can also be explained by incomplete lineage sorting, wherein the “correct” ribotype is masked by an incompletely sorted ribotype preferred by a possible PCR bias. However, the regions of the SSU gene targeted by the primers used for this study are very conservative, therefore a PCR bias can be introduced only due to different amplicon length and in our case the length of our amplicons across all of the samples does not show high variability (ca. 2%). We thus hypothesize that we are witnessing ongoing, yet incomplete speciation. This may or may not lead to completely isolated species within *H*. *serpula*; and from this reason we propose to treat the two morphologically separated entities (var. *serpula* and var. *parviverrucospora*) as varieties, not as species. This would concur with the moderate endemicity model [44–46]. A similar argument, assuming ongoing speciation, was put forward for a worldwide study using SSU genes in the planktonic foraminiferan *Globigerinella* sp., where incomplete separation of cryptic lineages was noted [71]. If our interpretation is valid, this would be the first evidence of ongoing speciation for myxomycetes.

**Table 3:** Analysis of molecular variance comparing each population among regions and among each clades

*Regions (temperate zone vs*. *Paleotropics vs*. *Neotropics)*				
Source of variation	d.f.	Sum of squares	Variance components	Percentage of variation
Among regions	2	3833.8	45.0	37.8
Within region	126	9326.6	74.0	62.2
Total	128	13160.5	119.8	
F_ST_ Fixation Index	0.38			
*Clades (clade 1 vs*. *clade 2 vs*. *clade 3)*				
Source of variation	d.f.	Sum of squares	Variance components	Percentage of variation
Among clades	2	7109.5	85.2	63.9
Within clade	126	6051.0	48.0	36.0
Total	128	13161.0	133.1	
F_ST_ Fixation Index	0.64			

The ancestral area reconstruction postulated *H*. *serpula* to originate in the Neotropic region (Fig 5). Several phylogeographic studies propose as well Neotropical origins for protists, such as *Leptomonas pyrrhocoris* (Trypanosomatidae, [72]), or *Badhamia melanospora* (Myxomycetes, [27]).

### Geographic patterns or ecological constraints?

The few available studies about geographic separation in myxomycete populations using molecular markers showed mixed patterns. No geographic separation at all was observed in populations of the dark-spored species *Lamproderma columbinum* in a limited sampling area (<50 km diameter) in deep ravines of Saxony, Germany [12]. Similarly, a worldwide study using mtDNA for *Didymium difforme* [73] did not reveal geographic differentiation, although the marker resolution may not be sufficient in the latter case. A study on the bright-spored myxomycete *Trichia varia* showed weak evidence for geographic separation of three reproductively isolated biospecies collected across Eurasia [30]; but one of these occurred much more common than the two others in a forest pocket in Northeastern Germany [26].

In our study, the correlation between genetic and geographic distance matrices (0.4667) indicated that geographical differentiation explains at least partly genetic variation in *Hemitrichia serpula*. For a widely distributed morphospecies like *H*. *serpula* geographic restriction may potentially lead to speciation, if mutations in mating-type genes governing amoebal compatibility become widespread in a regional population and cut it off from gene flow (see discussion in [20]). Such mutations should facilitate genotypic divergence, but with Kamono *et al*. [38] we must still assume a limited amount of gene flow between continents, mediated by long-distance dispersal. Apparently, some regions can serve as transitional zones. A good example is the temperate broadleaved forest of the Russian Far East, harboring ribotypes from clades 1 and 2. In accordance, the environmental niche models showed as well a niche overlap between clades 1 and 2 in this region (Fig 6).

The niche models (Fig 6) clearly showed that all three major clades overlap in the tropical ecozone. However, the putative biospecies represented by these clades may have different ecological requirements, with ribotypes from clade 3 showing a more restricted distribution (pantropical) than ribotypes of clades 1 and 2. Furthermore, the models suggested that ribotypes from clade 3 have an exclusive environmental niche only in tropical regions. Patterns like these of ribotypes in clade 3 (which showed 12 ribotypes exclusively occurring in Costa Rica and three ribotypes recorded only from Vietnam) and the fact that one ribotype from clade 1 was found once in Poland and many times in Far East of Russia would be better explained by dispersal limitations than environmental constraints. Nevertheless, it is important to note that these models only generate a probability map for the likeliness of occurrence of the ribotypes in each clade based on our available data and thus should not be seen as a truly predictive approach. The AUC values we generated come from presence-only data which makes them prone to bias. In addition, a simulation study [69] suggested having a minimum sample size of 13 independent samples for a widespread species. As usual for myxomycetes, we still lack information regarding the actual occurrence of *H*. *serpula* in many parts of the world (but see www.discoverlife.org). Similarly, Aguilar *et al*. [27] assumed a weak dispersal scenario explaining the niche models for two ribotype groups among the *Badhamia* population (although in this case possible reproductive isolation could not be demonstrated, since only one marker was used).

## Conclusion

This study demonstrates intraspecific variation in the morphologically clear-cut cosmopolitan myxomycete species *Hemitrichia serpula*. Distribution of ribotypes suggests limited gene flow between continents, which may lead to allopatric speciation caused by geographic barriers. The EF1A gene as a second marker indicates reproductive isolation between groups of ribotypes, which may be caused by incompatibilities in mating type genes, as demonstrated for Physarales in early cultivation experiments [19]. This would enable sympatric speciation processes. In our case, we found additional morphological evidence for speciation within *H*. *serpula*: the two most common varieties (var. *parviverrucospora* and var. *serpula*) were assigned to different putative biospecies, whereas one of the two rare varieties (var. *tubiglabra*) was likely to represent a maldeveloped specimen. However, heterogeneous (SSU) and heterozygous (EF1A) sequences together with the incomplete separation of morphotypes among major clades indicates speciation to be incomplete.

What do these data tell about myxomycete biogeography? Myxomycetes are ubiquitous in the sense that they are able to reach every suitable habitat on earth, at least considering a geological time scale. Consequently, many morphospecies show wide, sometimes cosmopolitan, but local distribution patterns, as shown for *Barbeyella minutissima* [37]. Such patterns of global ubiquity and local endemism are not uncommon for terrestrial protists, as demonstrated for the algal genus *Klebsormidium* [74]. Following this line of thought, the “everything is everywhere” hypothesis [42] for protist biogeography is met, at least if the limitation “but the environment selects” is regarded as well [43, 75]. However, with a more differentiating species concept (biospecies) that looks deeper into the molecular diversity of a widely distributed species, it becomes apparent that the gene flow mediated by possible long-distance dispersal of spores, which enables myxomycetes to fill out their whole potential distribution range, is not strong enough to prevent differentiation in regional gene pools, which may be the first step towards speciation.

## Supporting information

S1 File (Microsoft Excel 2010). Database including collection number, geographic coordinates, SSU ribotype (for all 135 specimens), EF1A genotype (for 30 selected specimens), and morphotype for the investigated accessions of *Hemitrichia serpula*. (XLS)

S2 File (fasta file). Alignment of partial SSU sequences for 135 specimens (making 138 sequences with three specimens being heterogeneous for SSU) used in this study.
(FAS)

S3 File (fasta file). Alignment of partial EF1A sequences of 30 specimens (30 sequences) used in this study. Specimen sc28067 shows a heterozygous sequence and is therefore listed twice.
(FAS)
